# The Election to the General Medical Council

**Published:** 1901-11-30

**Authors:** 


// # The Hospital

A Journal of

The Medical Sciences and Hospital Administration.

Vol. XXXI.—No. 792.] Edited by Sir Henry Burdett, K.C.B., and Solomon C. Smith, M.D., M.R.C.P. [November 30, 1901.

### THE ELECTION TO THE GENERAL MEDICAL COUNCIL.

The election of direct representatives on the General Medical Council is a duty laid upon the medical profession, and an obligation lies on every practitioner to use care in the exercise of his franchise. Still it cannot be said that any grave or burning issue depends upon his vote, and indeed the chief point of interest in the present election lies in the insight which the addresses of the candidates give as to the views upon certain current questions which are acceptable to the profession. So far as concerns the midwives question no one who considers the position now taken by the General Medical Council can believe that this will be altered in the slightest degree by any new light that the direct representatives can throw upon the subject. The Council has been asked for its opinion and has given it, and it is not to be expected that it will move from the position it has taken up, a position which, after all, is a very logical one. As voiced by its would-be representatives, however, we must take it that the medical profession believes that the solution of the question is to be found in what is practically the abolition of the midwives. They are to become obstetric nurses, they are to be employed and paid by the local sanitary authorities, they are to work under the direct supervision of medical practitioners, and these practitioners also are to be paid out of the rates! The whole affair is cut and dried, and nothing can be more complete. But is it seriously believed that any such scheme has the slightest chance of becoming law? Let us think for a moment of the sort of people we have to deal with, of the miserable fees which sanitary authorities think fit to offer to medical officers of health, of the starvation "wages" given by boards of guardians to their medical officers, and then ask ourselves whether any such public body is likely to saddle itself with the duty of paying for both doctor and nurse in cases which are commonly regarded by the people at large (*i.e.*, the electors) as only requiring the services of some neighbour who makes up in kindness for the general dirtiness of her attentions. Public opinion must advance wonderfully on this question, and must take a direction which it at present shows no tendency whatever to take before any such suggestion as that of the Manchester Guild comes within the range of practical politics. Yet we find doctors by the dozen pinning their faith upon it! Then in regard to the registration of nurses. Can anyone who takes an outside view of the matter believe for a moment that it would be possible to make "nursing" a closed profession; to register all those who are regarded as proper and fit persons for the work, and to prevent the rest from acting as nurses to the sick? This is a matter which touches the daily domestic life of every family, and one that cannot be decided on purely medical grounds. Moreover, it may fairly be asked how such a scheme would affect the provision of midwives or obstetric nurses for the poor? If the midwife is to be first a trained nurse, the cheapness of the midwife, which is her real *raison d'être* goes by the board. Take another point as to which the candidates, although not unanimous in regard to the extent to which they would go, seem at one in their aspirations—namely, the suppression of unqualified practice. From an inside and purely professional point of view this is a most admirable object, and wherever medical men foregather, it is a subject on which unanimity prevails. But, although "Down with quackery!" is easily said, all such work is quite outside the duties of the General Medical Council as constituted by law, and alterations of the law are not to be secured by any amount of talk in medical coteries. Legislation can only be obtained by those who are willing to rub shoulders with the world, and we ask again in regard to this question also Is our experience of the world so smooth and pleasant, and do we find outsiders so willing to accept our ex-cathedra utterances, as to make us regard it as probable that the legislature would under any circumstances make it a penal offence for an unregistered person to give or even to sell his advice or treatment in regard to any of the ills which flesh is heir to? Medical men who think that Parliament will ever suppress unqualified practice are, indeed, living in a fool's paradise.

What we have to do in regard to this election is to return good, strong, honest men without paying too much attention to the various panaceas for medical ills which they put forward as attractive to medical voters. But if the profession really wishes to raise its position and to have its grievances righted by legislation we must not depend too much on the action of the General Medical Council, but rather must endeavour, each in our own little circle, to influence those by whom laws are made, to render ourselves people of social and political importance, and not mere tinkerers of broken-down humanity; and whenever we discuss medico-political subjects we must not regard them so much as we have been in the habit of doing, from the inside, but must go out into the world and discuss them with our good friend the "man in the street." We shall probably be somewhat astonished at the laxity of his views in regard as to medical etiquette and at the very free and easy manner in which he deals with the gravest professional problems. Yet he is the law-maker—which must not be forgotten.

